# Serological testing of an equal-volume milk sample – a new method to estimate the seroprevalence of small ruminant lentivirus infection?

**DOI:** 10.1186/s12917-023-03599-z

**Published:** 2023-02-10

**Authors:** Adrian-Valentin Potârniche, Michał Czopowicz, Olga Szaluś-Jordanow, Agata Moroz-Fik, Marcin Mickiewicz, Kinga Biernacka, Lucjan Witkowski, Iwona Markowska-Daniel, Emilia Bagnicka, Constantin Cerbu, Diana Olah, Emilia Trif, Marina Spinu, Jarosław Kaba

**Affiliations:** 1 Department of Infectious Diseases and Preventive Medicine, Faculty of Veterinary Medicine, University of Agricultural Sciences and Veterinary Medicine Cluj-Napoca, Calea Manastur 3-5, 400372 Cluj-Napoca, Romania; 2 Division of Veterinary Epidemiology and Economics, Institute of Veterinary Medicine, Warsaw University of Life Sciences-SGGW, Nowoursynowska 159C, 02-776 Warsaw, Poland; 3 Department of Small Animal Diseases with Clinic, Institute of Veterinary Medicine, Warsaw University of Life Sciences-SGGW, Nowoursynowska 159C, 02-776 Warsaw, Poland; 4 Institute of Genetics and Animal Biotechnology, Polish Academy of Sciences, Postępu 36A, 05-552 Jastrzębiec, Magdalenka Poland

**Keywords:** Bulk-tank milk, Caprine arthritis-encephalitis, Competitive ELISA, Indirect ELISA, Within-herd seroprevalence

## Abstract

**Background:**

In cattle attempts to evaluate within-herd prevalence of various infectious and parasitic diseases by bulk-tank milk (BTM) testing with ELISA have been made with moderate success. The fact that BTM is composed of variable and unknown volumes of milk from individual lactating animals weakens the relationship between numerical result of the ELISA and the within-herd prevalence. We carried out a laboratory experimental study to evaluate if a pooled milk sample created by mixing an equal volume of individual milk samples from seropositive and seronegative goats, henceforth referred to as an equal-volume milk sample (EVMS), would allow for accurate estimation of within-herd seroprevalence of caprine arthritis-encephalitis (CAE) using 3 different commercial ELISAs. By mixing randomly selected milk samples from seronegative and seropositive goats, 193 EVMS were created – 93 made of seronegative samples and 100 with the proportion of seropositive individual milk samples (EVMS_%POS_) ranging from 1 to 100%. EVMS_%POS_ could be considered as a proxy for the within-herd seroprevalence. Then, OD of EVMS (OD_EVMS_) of the 193 EVMS was measured using 3 commercial ELISAs for CAE – 2 indirect and 1 competitive.

**Results:**

The cut-off values of OD_EVMS_ indicating SRLV infection were determined. The regression functions were developed to link OD_EVMS_ with EVMS_%POS_. A significant monotonic relationship between OD_EVMS_ measured with 2 commercial indirect ELISAs and EVMS_%POS_ was identified. Two regression models developed on this basis described approximately 90% of variability and allowed to estimate EVMS_%POS_, when it was below 50%. High OD_EVMS_ indicated EVMS_%POS_ of > 50%.

**Conclusion:**

Our study introduces the concept of serological testing of EVMS as a method of detecting SRLV-infected herds and estimating the proportion of strongly seropositive goats. Further field studies are warranted to assess practical benefits of EVMS serological testing.

**Supplementary Information:**

The online version contains supplementary material available at 10.1186/s12917-023-03599-z.

## Background

Caprine arthritis-encephalitis (CAE) caused by small ruminant lentivirus (SRLV) is one of the major health problems of goat population worldwide. SRLV infection is lifelong and in a proportion of goats progresses into a symptomatic form, with chronic arthritis being the most common clinical manifestation. Antibodies specific to SRLV are usually produced 4 to 12 weeks post infection, rarely later [1, 2], and remain detectable for life although their levels are known to fluctuate [3–5]. It makes serology the mainstay of CAE diagnostics [6, 7]. Serum testing is considered the optimal diagnostic modality for CAE control programs [8]. Testing of milk samples instead of blood eliminates animal stress as well as reduces costs associated with specimen collection. Therefore, several studies have evaluated diagnostic accuracy of serological milk testing [8–15]. Their results indicate high diagnostic accuracy and high agreement between qualitative results obtained on milk and serum. This makes milk testing a screening method worthy of consideration in dairy goat herds.

Regardless of the disease in question and the biological material used, serological screening of the herd is an expensive procedure as it requires a representative group of animals be properly selected and sampled [16]. This fact has given rise to the idea of serological testing of a bulk tank milk (BTM) sample. This approach is currently used in cattle for screening herds for various viral [17–20], bacterial [21, 22], and parasitic diseases [23–28]. In goats, serological BTM testing has so far been evaluated in terms of CAE, caseous lymphadenitis [29], toxoplasmosis [30], paratuberculosis [31], and Q fever [32, 33]. Estimations of diagnostic accuracy of serological BTM testing vastly depend on the reference standard used. Generally, in the aforementioned studies diagnostic specificity usually outraced sensitivity [29] and higher sensitivities were observed when serological rather than molecular reference standards were used [31]. Serological BTM testing for CAE was shown to be moderately accurate in detecting herds with at least 2% within-herd seroprevalence with the area under ROC curve of 80%. At an optimal cut-off value the procedure was roughly 73% sensitive and 84% specific [29].

The intensity of color reaction in ELISA performed on BTM sample, both raw and corrected optical density (OD), is known to correlate with the within-herd prevalence of infections in lactating individuals. As a consequence, quantitative ELISA results obtained on BTM samples have been used to estimate the within-herd prevalence of infections with bovine viral diarrhea virus (BVDV) [34, 35], bovine herpesvirus type 1 (BHV-1) [36–38], bovine leukemia virus (BLV) [39], and *F. hepatica* infection in cattle [23, 24], and *T. gondii* infection in goats [30]. However, the contribution of individual animals to the seroreactivity of a BTM sample depends on the volume of milk they yield and the concentration of antibodies in individual milk samples. As these two variables are not only beyond examiner’s influence but usually also remain unknown, estimations made on BTM samples have been shown to be rather imprecise. The former source of variability can be easily eliminated by creating an artificial milk sample by mixing an equal volume of milk from each animal. A recent study has shown that serological testing of this type of pooled milk samples from only 10 first-lactation cows in a herd yields more accurate result than BTM testing in terms of lungworm diagnosis [40]. This study, however, evaluated qualitative test results. On the other hand, Mazzei et al. [41] showed that the correlation between corrected OD of a pooled milk sample and the share of sheep milk seropositive for maedi-visna disease in this sample was almost perfect (coefficient of determination (R^2^) = 0.98). However, in this study the second source of variability was also removed by constant diluting a milk sample from the same seropositive sheep in the same seronegative milk sample (made by pooling BTM samples from 3 seronegative herds). Such perfect conditions preclude using the regression formula derived in their article to estimate the within-herd prevalences in field conditions.

Therefore, we carried out a laboratory experimental study to evaluate if testing a pooled milk sample created by mixing an equal volume of individual milk samples selected randomly from seropositive and seronegative goats (henceforth referred to as an equal-volume milk sample, EVMS) using three different commercial ELISAs would accurately estimate the proportion of milk samples coming from SRLV-seropositive goats.

## Results

The list of 193 EVMS along with their optical density (OD_EVMS_) and the proportion of seropositive individual milk samples in EVMS (EVMS_%POS_) are presented in Table S1.

### Negative EVMS

OD_EVMS_ of sp-iELISA ranged from 0.052 to 0.143 with the arithmetic mean (SD) of 0.070 (0.021) and significantly non-normal (*p* < 0.001) and right-hand skewed distribution (coefficient of skewness [CoS] = 2.07, CI 95%: 1.58 – 2.56). The cut-off value of OD_EVMS_ above which EVMS should be classified as positive was set at 0.15. One positive EVMS (with EVMS_%POS_ = 1%) overlapped with negative EVMS and therefore 99 EVMS with OD_EVMS_ ≥ 0.15 were further investigated for the relationship between OD_EVMS_ and EVMS_%POS_.

OD_EVMS_ of TM/CA-iELISA ranged from 0.118 to 0.928 with the arithmetic mean (SD) of 0.290 (0.141) and significantly non-normal (*p* < 0.001) and right-hand skewed distribution (CoS = 2.09, CI 95%: 1.60 – 2.58). The cut-off value of OD_EVMS_ above which EVMS should be classified as positive was set at 0.93. No positive EVMS overlapped with negative EVMS and therefore all 100 EVMS with OD_EVMS_ ≥ 0.93 were further investigated for the relationship between OD_EVMS_ and EVMS_%POS_.

OD_EVMS_ of SU-cELISA ranged from 0.476 to 0.855 with the arithmetic mean (SD) of 0.719 (0.092) and significantly non-normal (*p* < 0.001) and left-hand skewed distribution (CoS = -1.04, CI 95%: -1.53 – -0.55). The cut-off value of OD_EVMS_ below which EVMS should be classified as positive was set at 0.47. Six positive EVMS (with EVMS_%POS_ from 1 to 6%) overlapped with negative EVMS and therefore 94 EVMS with OD_EVMS_ ≤ 0.47 were further investigated for the relationship between OD_EVMS_ and EVMS_%POS_.

The aforementioned cut-off values of OD_EVMS_ ensured 100% diagnostic specificity (95% confidence interval [CI 95%]: 96.0% – 100%) of EVMS testing. However, diagnostic sensitivity of EVMS testing certainly was lower and was mainly affected by the analytical sensitivity of the ELISA which was not evaluated in this study. Therefore, EVMS testing yields results with high positive predictive value and positive result is highly trustworthy. However, EVMS testing should never be used for ruling SRLV infection out as the negative predictive value remains unknown. The study showed that low EVMS_%POS_ had OD_EVMS_ overlapping with OD_EVMS_ from negative EVMS.

### sp-iELISA on positive EVMS

**Fig. 1:**
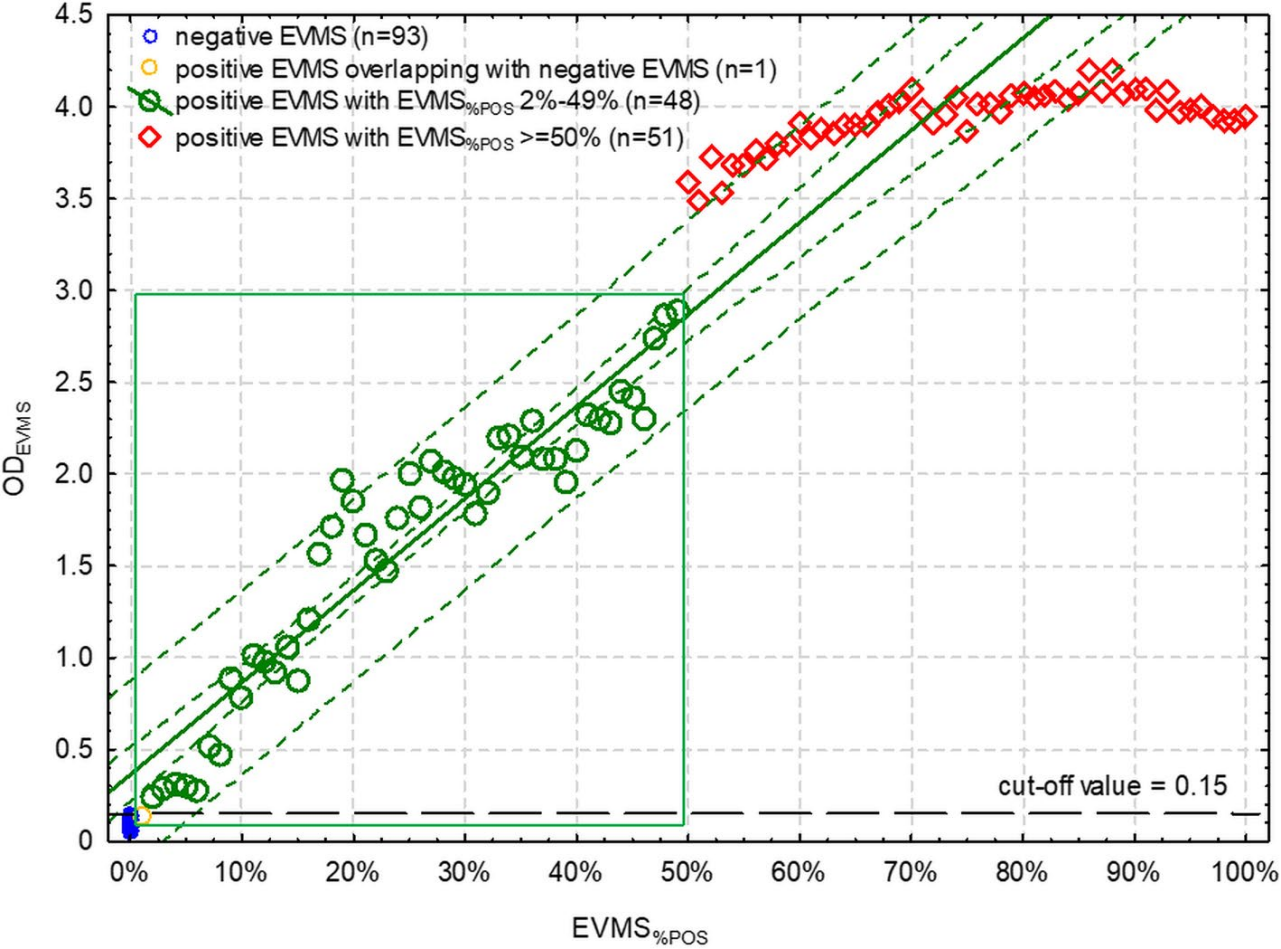
The relationship between the proportion of individual seropositive milk samples in the equal-volume milk sample (EVMS_%POS_) and optical density of EVMS (OD_EVMS_) in the indirect ELISA based on a mixture of synthetic viral peptides (sp-iELISA). The inner green broken line is a 95% confidence interval of the linear regression (green solid line). The outer green broken line is a 95% prediction interval. Green rectangle depicts the range of OD_EVMS_ within which the model allows for estimation of EVMS_%POS_. Black broken line is a cut-off separating positive and negative EVMS

The scatter plot showed a distinction in OD_EVMS_ between 99 samples with different EVMS_%POS_ (Fig. 1). OD_EVMS_ showed a gradual increase that was linearly linked to increasing EVMS_%POS_ (*r* = 0.946, CI 95%: 0.905 – 0.970; *p* < 0.001). This trend could be observed up to the EVMS_%POS_ of 50%, where the linear trend became significantly weaker (*r* = 0.725, CI 95%: 0.562 – 0.834; *p* < 0.001). At EVMS_%POS_ ≥ 50% OD_EVMS_ was > 3.0.

The EVMS model based on sp-iELISA and applied to OD_EVMS_ between 0.15 and 3.0 fit the data well (F_1,46_ = 390.8, *p* < 0.001) and was described by the following equation:

$${\mathrm{OD}}_{\mathrm{EVMS}}=0.364+5.015\times {\mathrm{EVMS}}_{\mathrm{\%POS}}\leftrightarrow {\mathrm{EVMS}}_{\mathrm{\%POS}}=\frac{{\mathrm{OD}}_{\mathrm{EVMS}}-0.364}{5.015}$$

Its parameters are given in Table 1 and R^2^ was 0.895. Standardized residuals were normally distributed (*p* = 0.213) and homoscedasticity was retained (*p* = 0.097).

**Table 1:** Parameters of two equal-volume milk sample (EVMS) models based on optical density (OD) measured using two indirect ELISAs

Variable	Regression coefficient (standard error)	95% confidence interval of the regression coefficient	*p*-value
EVMS model based on sp-iELISA			
Intercept	0.363 (0.074)	0.0.215 – 0.512	-
EVMS_%POS_	5.015 (0.254)	4.504 – 5.526	< 0.001
EVMS model based on TM/CA-iELISA			
Intercept	4.477 (0.055)	4.367 – 4.587	-
ln(EVMS_%POS_)	0.719 (0.029)	0.661 – 0.778	< 0.001

ELISA used: sp-iELISA* –* Indirect ELISA based on a mixture of synthetic viral peptides, TM/CA-iELISA* – *Indirect ELISA based on recombinant transmembrane and capsid proteins

The relationship between OD_EVMS_ measured using sp-iELISA and EVMS_%POS_ in the entire range of possible OD values was as follows:

$$\left\{\begin{array}{c}{\mathrm{if\ OD}}_{\mathrm{EVMS}}<0.15\rightarrow{\mathrm{EVMS\ negative\ or\ low\ EVMS}}_{\%\mathrm P\mathrm O\mathrm S}\\{\mathrm{if\ OD}}_{\mathrm{EVMS}}\epsilon\langle0.15,3.0\rangle\rightarrow{\mathrm{EVMS}}_{\%\mathrm P\mathrm O\mathrm S}=\frac{{\mathrm{OD}}_{\mathrm{EVMS}}-0.364}{5.015}\\{\mathrm{if\ OD}}_{\mathrm{EVMS}}>3.0\rightarrow{\mathrm{EVMS}}_{\%\mathrm P\mathrm O\mathrm S}\geq50\%\end{array}\right.$$

### TM/CA-iELISA on positive EVMS

**Fig. 2:**
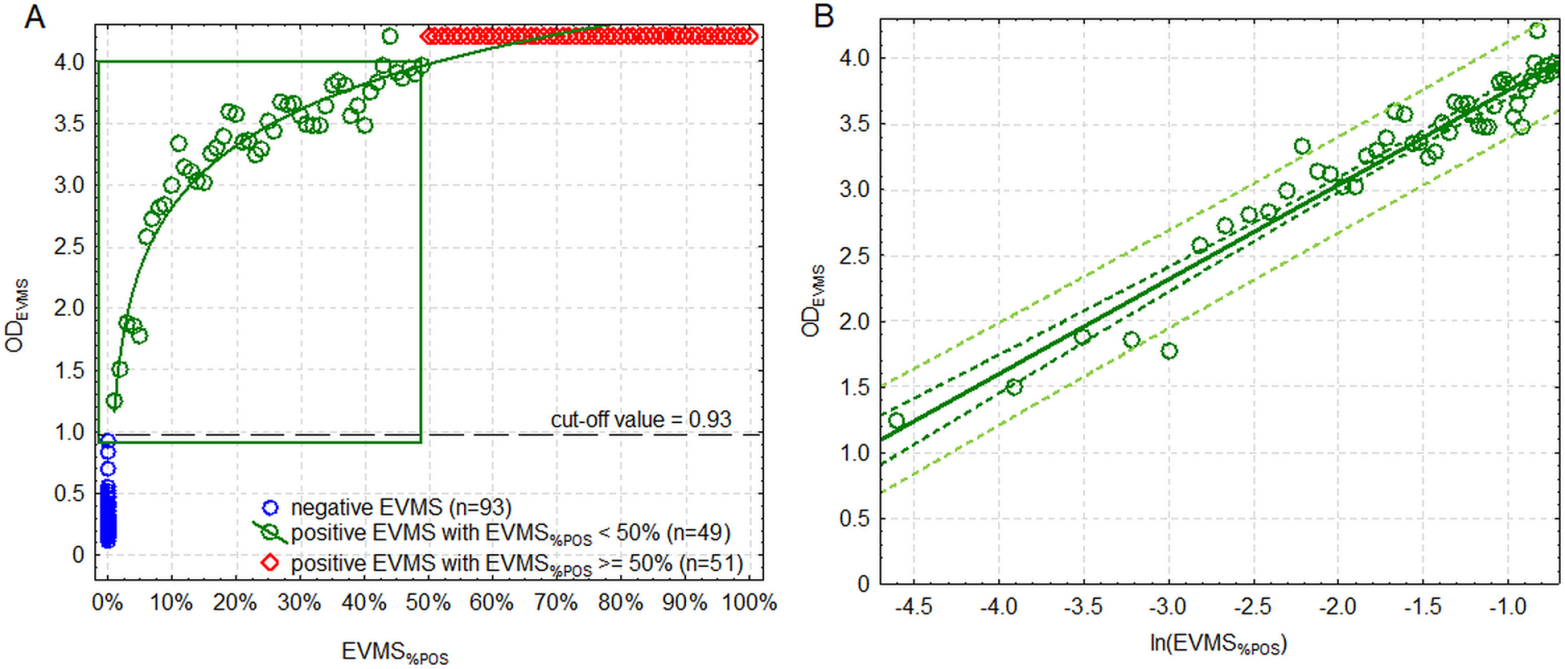
**A** (left): The relationship between the proportion of seropositive individual milk samples in the equal-volume milk sample (EVMS_%POS_, showed in logarithmic transformation) and optical density of EVMS (OD_EVMS_) in indirect ELISA based on recombinant transmembrane and capsid proteins (TM/CA-iELISA). Green rectangle depicts the range of OD_EVMS_ in which the model allows for estimation of EVMS_%POS_; **B** (right): Linearized regression function from the range presented in a green rectangle in the Fig. 2 A. Dark green broken line is a 95% confidence interval of the linear regression (green solid line). Light green broken line is a 95% prediction interval. Black broken line is a cut-off separating positive and negative EVMS

Also, in terms of this ELISA, the scatter plot showed a distinction in OD_EVMS_ between samples with different EVMS_%POS_ (Fig. 2). No positive EVMS overlapped with negative ones. Then, OD_EVMS_ showed a gradual logarithmic increase that was linked to increasing EVMS_%POS_ (after linearization using a logarithmic transformation of EVMS_%POS_: *r* = 0.964, CI 95%: 0.937 – 0.980). The logarithmic relationship could be observed up to the EVMS_%POS_ of 50% where the trend disappeared and the function became constant with OD_EVMS_ > 4.0.

To illustrate the relationship between OD_EVMS_ and EVMS_%POS_, EVMS_%POS_ from the range of 1% to 49% was logarithmically transformed (natural logarithm). The EVMS model based on TM/CA-iELISA and applied to OD_EVMS_ between 0.93 and 4.0 fit the data well (F_1,47_ = 610.4, *p* < 0.001) and was described by the following equation:

$${\mathrm{OD}}_{\mathrm{EVMS}}=4.477+0.791\times {\mathrm{ln\ EVMS}}_{\mathrm{\%POS}}\leftrightarrow {\mathrm{ln\ EVMS}}_{\mathrm{\%POS}}=\frac{{\mathrm{OD}}_{\mathrm{EVMS}}-4.477}{0.791}{\leftrightarrow \mathrm{EVMS}}_{\mathrm{\%POS}}={\mathrm{e}}^{\frac{{\mathrm{OD}}_{\mathrm{EVMS}}-4.477}{0.791}}$$

Its parameters are given in Table 1 and R^2^ was 0.927. Standardized residuals were normally distributed (*p* = 0.361) and homoscedasticity was retained (*p* = 0.425).

The relationship between OD_EVMS_ measured using TM/CA-iELISA and EVMS_%POS_ in the entire range of possible OD values was as follows:

$$\left\{\begin{array}{c}{\mathrm{if\ OD}}_{\mathrm{EVMS}}<0.93\rightarrow\mathrm{EMVS\ potentially\ negative}\\{\mathrm{if\ OD}}_{\mathrm{EVMS}}\mathrm\epsilon\langle0.93,4.0\rangle\rightarrow{\mathrm{EVMS}}_{\%\mathrm P\mathrm O\mathrm S}=\mathrm e^\frac{{\mathrm{OD}}_{\mathrm{EVMS}}-4.477}{0.791}\\{\mathrm{if\ OD}}_{\mathrm{EVMS}}>4.0\rightarrow{\mathrm{EVMS}}_{\%\mathrm P\mathrm O\mathrm S}\geq50\%\end{array}\right.$$

### SU-cELISA on positive EVMS

**Fig. 3:**
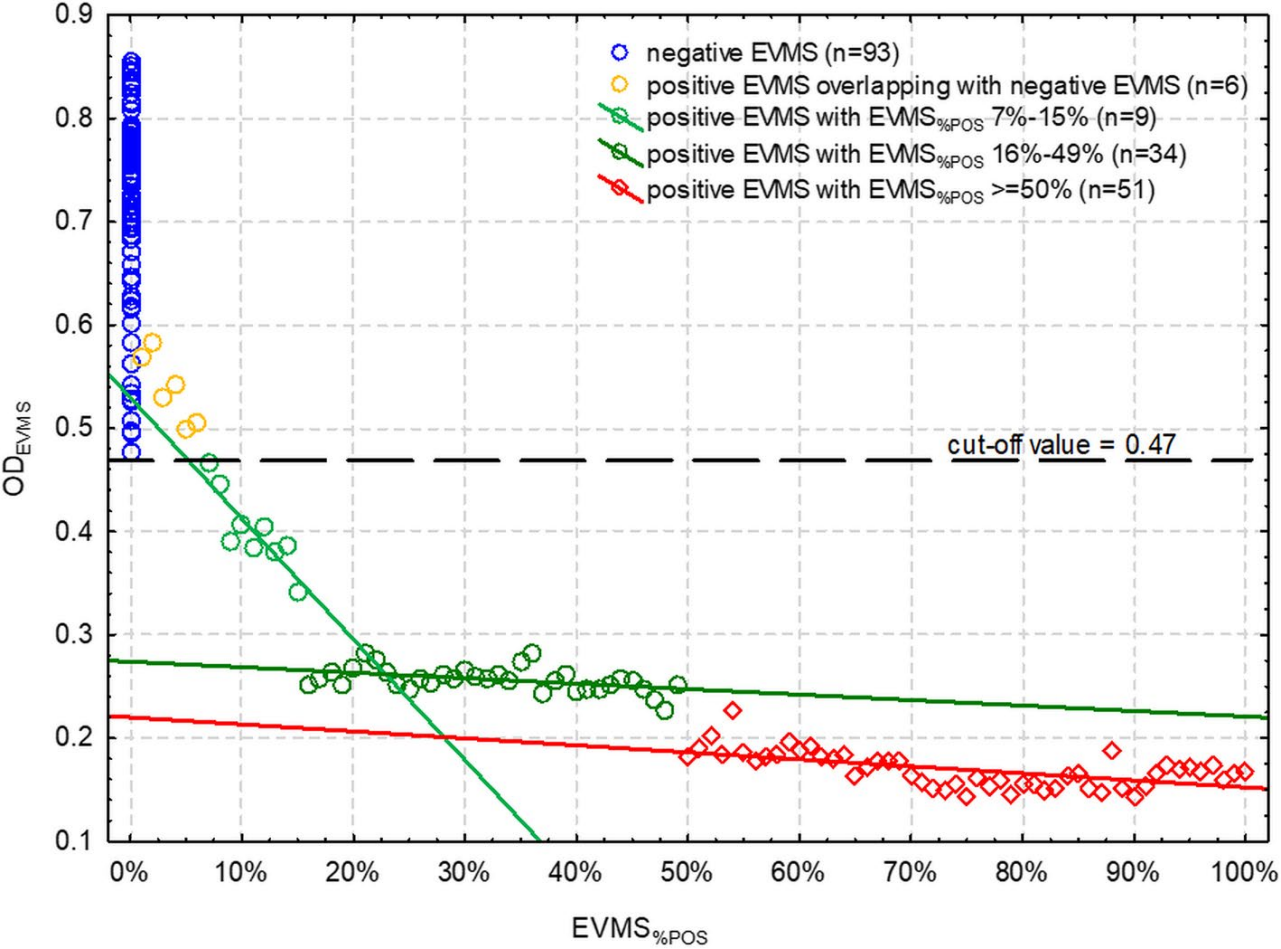
The relationship between the proportion of seropositive individual milk samples in the equal-volume milk sample (EVMS_%POS_) and the optical density of EVMS (OD_EVMS_) in competitive ELISA based on surface glycoprotein antigen (SU-cELISA). Black broken line is a cut-off separating positive and negative EVMS

As in the case of the other ELISAs, the scatter plot showed a distinction in OD_EVMS_ between samples with different EVMS_%POS_ (Fig. 3). Then, OD_EVMS_ showed a gradual decrease that was linearly linked to increasing EVMS_%POS_ (*r* = -0.871, CI 95%: -0.973 – -0.491; *p* = 0.002), which, however, could be observed only up to the EVMS_%POS_ of 15%. Then, the linear trend turned significantly weaker (*r* = -0.454, CI 95%: -0.687 – -0.137; *p* = 0.007). Such a trend was observed up to the EVMS_%POS_ of 49%, and then it slightly changed but remained similarly weak (*r* = -0.584, CI 95%: -0.740 – -0.368; *p* < 0.001). A very narrow range of EVMS_%POS_ (up to 15%) in which the relationship between OD_EVMS_ and EVMS_%POS_ followed a measurable trend indicated that SU-cELISA was unsuitable for estimation of EVMS_%POS_. As a result, it could not be used for estimation of the within-herd seroprevalence on the basis of EVMS.

## Discussion

Our study shows that a milk sample prepared by pooling equal volume of individual milk samples from all lactating animals in a herd, called by us an equal-volume milk sample (EVMS), is characterized by the presence of a very strong monotonic relationship between the proportion of individual milk samples from goats seropositive for SRLV infection used for preparation of this EVMS (EVMS_%POS_) and its OD value measured using a commercial indirect ELISA for CAE (OD_EVMS_). This relationship was observed despite the fact that milk samples were selected based on serological testing of respective serum samples not milk samples themselves and the agreement between OD values in these two materials is moderate, not only in CAE [8] but also in other infections [24, 42]. The strong monotonic relationship applies, however, only to a part of the range the two variables can take.

Our results offer a chance to use OD_EVMS_ not only to classify a herd as seropositive or seronegative but also to estimate the proportion of seropositive lactating goats in a herd. This concept is very tempting as practical benefits from quantitative evaluation of a herd status based on testing only one sample are undeniable. Hope for this has encouraged many scientific teams to attain this goal by testing a BTM sample [34–39]. However, despite significant correlations and R^2^ ranging from 0.7 to 0.85 a considerable spread of OD values from the regression line could be observed in all these studies. Moreover, the higher within-herd prevalence was the further OD values tended to be located from the regression line. In our study we observed the same trend as well as differences in the character of the relationship in subsequent ranges of the dependent variable (i.e. EVMS_%POS_). In fact, the significant and strong correlation between the two variables was present in our study only for EVMS_%POS_ < 50%. Within this range approximately 90% of variability could be explained by EVMS models. Above this value, the regression line became more or even completely horizontal in the case of sp-iELISA and TM/CA-ELISA, respectively. Therefore, the relationship between OD_EVMS_ and EVMS_%POS_ could not be described using a single function for the entire range of values of EVMS_%POS_ (0% to 100%). Interestingly, a similar phenomenon could be noticed after careful inspection of scatter plots presented in many previous studies on BTM in bovine viral infections [34, 36–39]. In these studies, exponential, square root, and logarithmic functions were used to link the two variables in the whole range of their possible values. However, our observations suggest that OD values correlate significantly only with a limited range of values the analyzed variables may take. Therefore, we think it is pointless to try to construct a single model predicting the entire range of EVMS_%POS_. Above a particular value, which in our study seemed to be 50%, the EVMS_%POS_ cannot be precisely estimated which is certainly a limitation of EVMS serological testing. In our opinion, the same limitation applies to most of so far published studies aiming to predict within-herd prevalence on the basis of OD value of BTM sample. Interestingly, this effect was absent in studies investigating this BTM testing in parasitic diseases [23, 24].

The regression equations we derived are only an illustration of the relationships observable in certain predefined and controlled laboratory conditions. It is unlikely that they can be directly used to estimate within-herd prevalences of CAE in field conditions. This is because EVMS_%POS_ is only a very simplified proxy for the within-herd prevalence. First of all, it was made by mixing milk samples from seronegative and strongly seropositive goats while not all SRLV infected goats are strongly seropositive. In fact, the levels of antibodies against SRLV are known to fluctuate [3–5] which is likely to blur the differences between situations in which 1 strongly seropositive goats or several weakly seropositive goats are present in a herd. The study of Mazzei et al. [41] shows that when the variability coming from different antibody concentrations in individual milk samples is eliminated by using still the same positive and negative milk sample to prepare milk dilutions, the correlation between OD value and proportion of seropositive parts of milk in the pooled sample is virtually perfect. However, such a situation is purely theoretical and does not correspond to field conditions. Further studies should answer the question about the magnitude of influence this factor has on the estimations based on OD_EVMS_. Secondly, we artificially created EVMS based on 100 individual milk samples. The influence of a single goat’s seroreactivity on the seroreactivity of EVMS was probably relatively smaller than if EVMS was based on 10 milk samples. However, the magnitude of this effect remains unknown and cannot be reliably predicted by extrapolation of our results.

Another drawback to our study is the unknown analytical sensitivity of the method. Commercial indirect ELISAs used in our study allowed to detect the presence of 1 (TM/CA-iELISA) or 2 (sp-iELISA) seropositive individual milk samples out of 100. This could falsely suggest that EVMS serological testing might serve as a screening method. It has to be emphasized that the minimum amount of antibodies that can be detected by the ELISAs in EVMS (limit of detection) is unknown. Therefore, no conclusions can be drawn from the fact only 1 or 2 positive EVMS of the lowest EVMS_%POS_ overlapped with negative EVMS. EVMS with e.g. 10% of positive individual milk samples could have also overlapped with negative EVMS if these positive milk samples had contained less antibodies. Testing EVMS may only indicate the seropositive status of the herd but not the seronegative status. The same applies to BTM testing. The freedom from disease can only be confirmed in properly-designed and properly-conducted disease surveys [16]. Our observation that SU-cELISA performed worse than the two indirect ELISAs is probably at least partly associated with lower analytical sensitivity of EVMS testing with this ELISA. SU-cELISA is based on a single SRLV antigen so it is able to capture only antibodies against SU, leaving anti-CA and anti-TM antibodies unattended. Therefore, is likely to detect fewer positive EVMS as it has also been shown in terms of individual milk samples [15] and serum [43].

The fact that a significant correlation between OD value of BTM samples and within-herd prevalence has been observed in many previous studies acts in favor of the concept of EVMS testing. EVMS overcomes one crucial weakness of BTM i.e. an inequality of milk sample volumes contributed by individual lactating females, which is likely to considerably lower the variability in predictive models. A concept close to EVMS was proposed and evaluated by McCarthy et al. [40]. They created pooled milk samples from 10 randomly selected heifers in a cattle herd. A diagnostic accuracy of serological test for lungworm infection carried out on this sample proved to be significantly higher than on BTM sample. Their study shows highly probable advantage of pooling equal volumes of milk over BTM in which contribution of individual animals to the total volume is unknown. Unfortunately, an attempt to estimate the within-herd seroprevalence was not made in this study. EVMS combines advantages of the pooled milk sample from the representative number of animals in a herd (equal representation of animals tested) and the BTM sample (representation of all lactating animals). Obviously, like BTM, EVMS does not include males, kids, and females before the first parturition, as well as goats in a dry off period. However, especially in goats, it is unlikely to considerably interfere with the assessment of the herd status as males constitute only a small part of a dairy herd (usually a few bucks are kept separately from females and can be tested individually) and goats usually are bred seasonally. As a result, most of lactating does are in a similar stage of lactation which eliminates a factor considered as a potential source of variability in BTM testing of dairy cattle herds [37, 44]. Moreover, the optimal moment for herd screening may easily be chosen. Modern milking parlors enable rapid and simple collection of an individual milk sample from the bucket (recording jar). As EVMS is prepared just by mixing such individual milk samples at equal volume, it adds very little work to farmer’s daily schedule. Compared to collecting individual blood or milk samples directly from individual animal’s vein or udder it not only saves money, time, and labor, but also spares animals additional stress.

## Conclusions

Our study introduces the concept of serological testing of an equal-volume milk sample (EVMS) as a method of detecting SRLV-infected herds and estimating the proportion of strongly seropositive goats. Analogically to BTM, EVMS ensures the representation of all lactating animals in the herd, meanwhile eliminating the variability associated with different volumes of milk yielded by each animal. Our study demonstrates that in terms of SRLV infection a significant monotonic relationship between OD_EVMS_ and EVMS_%POS_ is observed only for a part of the range of values these variables may take and only when indirect ELISAs are used. Further field studies are warranted to assess practical benefits of EVMS serological testing not only in terms of CAE but also other infectious and parasitic diseases in dairy animals.

## Methods

### Equal-volume milk samples

Numerical results (raw and corrected OD values) of 200 individual serum and milk paired samples were purposively selected from the database used in the previous study regarding performance of 3 commercial immunoenzymatic assays on individual milk samples [14]. Serum and milk (lactoserum) samples from individual goats had been collected for this study between April and August 2019 (the first half of lactation) and screened using two indirect ELISAs – ID Screen MVV-CAEV Indirect Screening test (ID.vet Innovative Diagnostics, Grabels, France) containing the panel of peptides from SRLV structural proteins – surface glycoprotein (gp135, SU), transmembrane glycoprotein (gp46, TM), and capsid protein (p25/p28, CA) (henceforth referred to as sp-iELISA), and IDEXX MVV/CAEV p28 Ab Screening (IDEXX Laboratories, Westbrook, ME, USA) based on recombinant TM and CA antigen (TM/CA-iELISA), and one competitive ELISA – Small Ruminant Lentivirus Antibody Test Kit, cELISA (VMRD, Pullman, WA, USA) coated with SU (SU-cELISA). The following criteria of sample selection were applied: One hundred paired serum and milk samples came from 100 goats that tested positive for the presence of antibodies to SRLV (seropositive goats) in the 3 ELISAs, and their corrected OD was at least twofold higher than the manufacturers’ cut-off values which were as follows: serum-to-positive control ratio (S/P%) = 50% in sp-iELISA, S/P% = 110% in TM/CA-iELISA, and percentage of inhibition (PI) = 35% in SU-cELISA. Another 100 paired serum and milk samples came from 100 goats that tested negative for the presence of antibodies to SRLV (seronegative goats) in the 3 ELISAs, and their corrected OD was at least twofold lower than the manufacturers’ cut-off values. Raw and corrected OD of paired 200 serum and milk samples are summarized in Table 2 and individual results can be found in Table S2.

**Table 2:** Numerical results of 3 ELISAs (optical density, OD) used in the study presented as the arithmetic mean ± standard deviation and range in parentheses

ELISA^a^	Seronegative goats (*n* = 100)				Seropositive goats (*n* = 100)			
	serum		milk		serum		milk	
	Raw OD	Corrected OD^b^ [%]	Raw OD	Corrected OD^b^ [%]	Raw OD	Corrected OD^b^ [%]	Raw OD	Corrected OD^b^ [%]
sp-iELISA	0.073 ± 0.038 (0.048 – 0.297)	2.3 ± 3.6 (-0.3 – 20)	0.065 ± 0.028 (0.045 – 0.240)	1.5 ± 2.5 (-0.5 – 15)	4.019 ± 0.248 (3.061 – 4.2)	353 ± 34 (286 – 429)	3.625 ± 0.690 (1.159 – 4.2)	316 ± 59 (111 – 403)
TM/CA-iELISA	0.160 ± 0.045 (0.100 – 0.330)	8.2 ± 4.8 (1.8 – 20)	0.169 ± 0.122 (0.051 – 0.933)	9.2 ± 12 (-2.7 – 78)	3.934 ± 0.297 (2.924 – 4.2)	376 ± 48 (302 – 474)	3.887 ± 0.443 (1.926 – 4.2)	372 ± 63 (179 – 474)
SU-cELISA	0.810 ± 0.156 (0.517 – 1.385)	-1.3 ± 12 (-45 – 9.9)	0.737 ± 0.179 (0.275 – 1.596)	7.6 ± 18 (-56 – 67)	0.189 ± 0.085 (0.062 – 0.383)	74 ± 12 (52 – 93)	0.286 ± 0.120 (0.059 – 0.585)	61 ± 15 (28 – 93)

^a^ ELISA used: sp-iELISA – indirect ELISA based on a mixture of synthetic viral peptides, TM/CA-iELISA – indirect ELISA based on recombinant transmembrane and capsid proteins, and SU-cELISA – competitive ELISA based on surface glycoprotein antigen

^b^ corrected optical density: sp-iELISA and TM/CA-iELISA – serum-to-positive control ratio (S/P%), SU-cELISA – percentage of inhibition (PI)

By mixing randomly selected milk samples from seronegative goats with randomly selected milk samples from seropositive goats, 193 equal-volume milk samples (EVMS) were created. Simple random method of selection with returning was performed using the RAND.BETWEEN() function in Microsoft Excel. For each EVMS, 10 μl of 100 individual milk samples were mixed to amount to the volume of 1 ml. Ninety three EVMS were made of individual milk samples from seronegative goats (negative EVMS). One hundred EVMS were made of individual milk samples from seropositive and seronegative goats combined at a ratio from 1:99 to 100:0 (positive EVMS). The proportion of seropositive individual milk samples out of 100 individual milk samples (EVMS_%POS_) increased by 1% from 1% to 100%.

### EVMS serological testing

Then, OD of EVMS (OD_EVMS_) of the 193 EVMS was measured using the 3 ELISAs. The ELISAs were performed according to manufacturers’ protocols. EVMS were diluted 1/2 in sp-iELISA and TM/CA-iELISA, and remained undiluted in SU-cELISA. These dilutions were chosen based on our previous study [15]. All other steps of the protocol remained unchanged compared to regular serum testing. OD_EVMS_ was measured at a wavelength of 450 nm (sp-iELISA and TM/CA-iELISA) or 620 nm (SU-cELISA) using the scanner (Epoch Microplate Spectrophotometer, BioTek, USA). OD_EVMS_ values exceeding the upper measuring limit of the scanner (OD > 4.0, OVERFLOW) were replaced with the value of 4.2.

### Statistical analysis

OD values were presented as the arithmetic mean, standard deviation (± SD), and range. Normality of distribution was assessed using the Quantile–Quantile scatter plots and the Shapiro–Wilk test, and the skewness of distribution was expressed using coefficient of skewness (CoS) with CI 95%. The cut-off value separating negative and positive EVMS was set at the maximum (in the case of indirect ELISAs) or minimum (in the case of SU-cELISA) OD_EVMS_ observed in negative EVMS rounded up or down, respectively, to the closest second decimal digit. Diagnostic specificity of EVMS testing was calculated as the number of negative EVMS below the cut-off value divided by 93 negative EVMS and the CI 95% was calculated using Wilson score method [45].

Linear correlation between OD_EVMS_ above the cut-off value and EVMS_%POS_ was determined using the Pearson product-moment correlation coefficient (r) and the CI 95% was calculated with the method of Altman et al. [45]. Strength of correlation was classified as follows: *r* = 0.00 to 0.19 – very weak; 0.20 to 0.49 – weak; 0.50 to 0.69 – moderate, 0.70 to 0.89 – strong, and 0.90 to 1.00 – very strong [46].

The relationship between EVMS_%POS_ (explanatory, independent variable, x) and OD_EVMS_ above the cut-off value (outcome, dependent variable, y) was investigated using the scatter plot and simple linear regression according to a general formula:

$$\mathrm{y}={\upbeta }_{0}+{\upbeta }_{1}\mathrm{x}+\upvarepsilon$$

where β_0_ was an intercept (an y value at which x = 0), β_1_ was a slope of the linear regression line, and $\upvarepsilon$ was a residual (error).

EVMS_%POS_ was considered an independent variable in this model because its values were fixed by the study design, and they had an influence on OD_EVMS_ which were, therefore, considered a dependent variable. Hence, the equation was as follows:

$${\mathrm{OD}}_{\mathrm{EVMS}}={\upbeta }_{0}+{\upbeta }_{1}\times {\mathrm{EVMS}}_{\mathrm{\%POS}}$$

Depending on the shape of relationship, x variable was either entered in raw or logarithmically transformed (natural logarithm with e basis) form. To predict EVMS_%POS_ based on OD_EVMS_ the equation was inversed as follows:

$${\mathrm{EVMS}}_{\mathrm{\%POS}}=\frac{{\mathrm{OD}}_{\mathrm{EVMS}}-{\upbeta }_{0}}{{\upbeta }_{1}}$$

Assumptions of the linear regression were evaluated as follows [47]: 1) shape of the relationship between x and y was assessed by visual inspection of the scatter plot and it was linearized by x transformation if necessary; 2) normality of residual distribution was assessed by visual inspection of the Quantile–Quantile scatter plots and using the Shapiro–Wilk test; 3) homoscedasticity was assessed by visual inspection of the standardized residual vs. predicted y variable plot and using Brown-Forsythe test on standardized residuals compared across 5 categories of EVMS_%POS_. The proportion of explained variability was evaluated by the coefficient of determination (R^2^). All statistical tests were two-tailed and a significance level (α) was set at 0.05. Statistical analysis was performed using TIBCO Statistica 13.3 (TIBCO Software Inc, Palo Alto, CA, USA).

## Supplementary Information

**Additional file 1: Table S1.** Raw and corrected optical densities (OD) of 193 equal-volume milk samples (EVMS) measured using 3 ELISAs along with the proportion of seropositive individual milk samples in the equal-volume milk sample (EVMS_%POS_).

**Additional file 2:** **Table S2.** Raw and corrected optical densities (OD) of 200 serum and milk samples used in the experiment measured using 3 ELISAs – indirect ELISA based on a mix of synthetic viral peptides (sp-iELISA), indirect ELISA based on recombinant transmembrane and capsid proteins (TM/CA-iELISA), and competitive ELISA based on surface glycoprotein antigen (SU-cELISA).

## Data Availability

The database is available from the authors (Adrian-Valentin Potârniche and Michał Czopowicz) on request.

## Abbreviations

α: Significance level
β_0_: Intercept of the linear regression line
β_1_: Slope of the linear regression line
BTM: Bulk-tank milk
CAE: Caprine arthritis-encephalitis
CI: Confidence interval
CoS: Coefficient of skewness
EVMS: Equal-volume milk sample
EVMS_%POS_: The proportion of seropositive individual milk samples in the equal-volume milk sample
OD: Optical density
OD_EVMS_: Optical density of the equal-volume milk sample
PI: Percentage of inhibition
r: Pearson product-moment correlation coefficient
R^2^: Coefficient of determination
SD: Standard deviation
S/P%: Sample-to-positive control ratio percentage
SRLV: Small ruminant lentivirus
SU-cELISA: Competitive ELISA based on surface glycoprotein of SRLV
TM/CA-iELISA: Indirect ELISA based on recombinant transmembrane and capsid protein of SRLV
sp-iELISA: Indirect ELISA based on a mixture of synthetic viral proteins
